# Cross-sectional association between frequency of vigorous physical activity and academic achievement in 214,808 adolescents

**DOI:** 10.3389/fspor.2024.1366451

**Published:** 2024-08-08

**Authors:** Xiaohui Zhang, Danqing Zhang, Xingyi Yang, Sitong Chen

**Affiliations:** ^1^ School of Physical Education, Suzhou University, Suzhou, China; ^2^ Centre for Mental Health, Shenzhen University, Shenzhen, China

**Keywords:** physical activity, academic performance, exercise, high intensity, schoolchildren

## Abstract

**Background:**

This study explores the intricate link between vigorous physical activity and academic achievement in adolescents. By analyzing data from the Health Behaviour in School-aged Children (HBSC) survey, it seeks to understand how engaging in high-intensity physical activities influences academic achievement.

**Methods:**

A comprehensive cross-sectional analysis was conducted on a vast dataset comprising 214,808 adolescents aged 11, 13, and 15 years. The frequency of vigorous physical activity was determined through self-reports, while academic achievement was assessed based on students’ self-perceptions, reflecting their understanding of teachers’ evaluations of their academic performance.

**Results:**

Adolescents who reported engaging in vigorous physical activity daily were more likely to report higher academic achievement. This positive correlation was consistent across various demographic groups, including different age cohorts and both genders, indicating a universal benefit of regular vigorous physical activity on perceived academic success.

**Conclusion:**

The findings of this study underscore the significant role that regular vigorous physical activity plays in the academic lives of adolescents. The correlation between daily vigorous physical activity and enhanced self-reported academic achievement suggests that encouraging high-intensity physical activities in schools could be a key strategy in boosting educational outcomes.

## 1 Introduction

Regular physical activity is important for children's development, as it enhances healthy growth and health outcomes, such as improving physical fitness, bone health and healthy weight status, while controlling type 2 diabetes, high blood pressure and cardiovascular diseases (1). Furthermore, physical activity benefits cognitive processes and meta-cognition, fostering engagement, academic performance, learning, and eligibility for higher education (2–5). Two studies revealed that children engaged in daily moderate physical activity during the nine years of compulsory education had higher eligibility for upper secondary school at ages 15–16, compared to those participating in physical activity 1–2 times weekly (3, 4). In a prospective controlled intervention trial, daily physical activity sessions from ages seven to fifteen were linked to a seven percent increase in boys’ eligibility for upper secondary school, but no significant change was observed for girls (3). A study by Mavilidi et al. added new knowledge to the literature, by showing that just 5 min of physical activity three times per week during maths lessons were associated with improved learning behaviour and performance (6).

Research indicates that physical activity positively influences the brain and nervous system, as evidenced that physically fit adolescents have larger brain volumes in key areas such as the hippocampus and basal ganglia, compared to their inactive peers (7). This is also replicated in older adults, where those who are physically active possess greater volumes of brain matter than less active ones (8). Electroencephalogram studies support these findings, revealing enhanced electro-cortical functions in physically active subjects (9). Animal research further indicates that physical activity promotes neural cell proliferation, survival, and long-term potentiation, essential for memory and learning (10). Additionally, physical activity increases catecholamines, which are crucial for learning (3, 10). Studies in neuroscience have found links between physical activity and changes in brain structure/function, including alterations in grey matter thickness (11), white matter integrity (12), and enhancements in attention, memory, and executive function (13–16). These neurobiological benefits are believed to contribute to improved academic achievement. Beyond these direct effects, physical activity also indirectly benefits cognition and academic performance through improvements in psychological factors such as self-esteem and mental health (3, 4). A comprehensive meta-analysis has confirmed the broad impacts of physical activity on various cognitive aspects in children aged 4–18, demonstrating its extensive influence on the learning process (17).

Researchers have extensively explored the relationship between physical activity and academic achievement in adolescents, focusing on its impact on both academic outcomes and cognitive functioning (18–20). Meta-analyses have consistently demonstrated a positive link between physical activity and cognitive performance, revealing significant benefits of physical activity on academic performance in children and adolescents (21). However, earlier studies primarily assessed the effects in preadolescent children through a few intervention studies, often involving longitudinal physical activity programs (22). More recent research includes randomized controlled trials investigating both acute and chronic physical activity programs and their influence on cognitive functions (23–25). While the association between physical activity, particularly moderate to vigorous, and academic achievement is generally positive (19, 20), the findings are not always consistent. Some studies also reported non-significant associations (26, 27) or even negative impacts (19, 28) of physical activity on academic performance. It is noteworthy that most research concentrates on moderate to vigorous physical activity, with very few specifically examining the individual effects of vigorous physical activity on academic achievement in adolescents.

While the current body of literature offers valuable insights into the association between physical activity intensity and academic performance, a significant research gap is apparent, particularly when investigating the influence of vigorous physical activity on academic outcomes among adolescents within large sample size. Previous studies, including those exploring the effects of the High-Intensity Interval Training (HIIT) interventions, have shown promising enhancements in executive functions (29–31). However, these interventions were often characterized by small sample size, and a lack of comprehensive exploration of academic outcomes. Tomporowski's 2003 meta-analysis focused on the impact of acute bouts of physical activity on information processing, revealing an inverted U-shaped relationship between acute physical activity and cognitive performance. This underscored the potential for decreased cognition with physical fatigue, aligning with the literature suggesting that over-arousal may be detrimental to performance. In contrast, certain researchers, including Chang, Labban, Gapin, and Etnier (2012), underscored the pivotal role of exercise intensity (32). They proposed that low-intensity PA immediately facilitates cognitive processes after exercise, while high-intensity work may induce fatigue and subsequent cognitive degradation, with potential improvement after physiological recovery. The present study, recognizing the interconnected nature of physical activity intensity as closely linked variable, cautiously presents results indicating a potential positive association between vigorous physical activity and increased math scores. This finding aligns with earlier literature reporting similar outcomes after high-intensity physical activity interventions, as demonstrated by Castelli et al. (33). However, a critical research gap exists in exploring vigorous physical activity and its specific impact on academic outcomes among adolescents within large and diverse sample sizes. Future research endeavours should prioritize addressing this gap to provide a more comprehensive understanding of how vigorous physical activity influences cognitive functions and academic performance. Such investigations are pivotal in guiding evidence-based strategies to optimize the positive impact of physical activity on academic outcomes among adolescents.

The aim of the present study therefore was to investigate the association between frequency of vigorous physical activity and academic achievement in large sample size of adolescents.

## 2 Methods

### 2.1 Study design

This study used a cross-sectional approach, utilising data from the 2014 Health Behaviour in School-aged Children (HBSC) study conducted in Europe and North America. The HBSC survey, a collaborative initiative led by the World Health Organisation (WHO), gathered information from adolescents with a four-year interval. Adolescents provided insights into their health status through self-administered questionnaires distributed within classroom settings. Individual countries had the flexibility to categorise their samples, ensuring diverse representation based on factors such as geographical location, ethnicity, and school type. The recruitment process employed a methodical multistage stratified cluster randomised sampling approach. The survey was conducted at various points during the academic year, aligning with national school schedules and resulting in samples reflecting mean ages of 11.5, 13.5, and 15.5 years. Participants completed the HBSC questionnaire under the supervision of teachers, school nurses, or other designated staff, following standardised instructions (34, 35). Ultimately, this analysis included 214,808 adolescents aged 11, 13, and 15 years.

### 2.2 Measures

#### 2.2.1 Frequency of vigorous physical activity

The frequency of vigorous physical activity was evaluated using the following question: “During non-school hours, how often do you usually exercise in your free time so much that you get out of breath or sweat?” Responses were: “Every day”, “4–6 times a week”, “2–3 times a week”, “Once a week”, “Once a month”, “Less than once a month”, “Never”. Prior studies have demonstrated that the frequency of vigorous physical activity item from the HBSC study has acceptable reliability, with ICC values ranging from 0.62 to 0.71, and fair validity, demonstrated by a correlation with maximal oxygen consumption (VO2max) of *r* = 0.33, in students from various countries (36–39).

#### 2.2.2 Academic performance

Academic performance was gauged with a single question: “In your opinion, what does your class teacher(s) think about your school performance to your classmates?” Response options included: “very good”, “good”, “average”, and “below average”. Self-reported academic performance has been confirmed as an acceptable and valid measure in the previous research (40).

#### 2.2.3 Covariates

Demographic information, including age, gender, well off, self-rated health, screen time (i.e., watching TV, playing game, using computer, breakfast, soft drinks, sweets, vegetables, fruits and alcopops consumption as well as smoking were collected from study participants using a self-reported questionnaire.

### 2.3 Statistical analysis

All analyses were conducted using IBM SPSS version 28.0. The descriptive characteristics of participants were reported as percentage (%). Ordered logistic regression models were employed to examine the association between vigorous physical activity and weight status across the overall sample, as well as separately for boys and girls. Participating in vigorous physical activity every day was set as the reference group. All models were adjusted for age, gender, well off, self-rated health, screen time, eating habits, alcopops consumption, and smoking (except for sex in the overall sample). The statistical significance level was set as *p* < 0.05 in this study.

## 3 Results

A pooled sample of 214,808 adolescent (boy: 49.2%) with aged 11, 13 and 15 years old were extracted from the HBSC survey. The prevalence of good and very good academic achievement was 43.2% and 24.0%. Results indicate the proportion of study participants who engaged in frequency of vigorous physical activity at different levels. 19.2%, 24.4%, 29.2%, 13.1%, 3.8% and 4.7% of study participants reported engaging in frequency of vigorous physical activity every day, 4–6 times a week, 2–3 times a week, once a week, once a month and less than once a month, respectively. More details can be found in Table 1.

**Table 1:** Sample characteristics of this study.

Variables	Proportion	Variables	Proportion
Age		Smoking	
11 years old	32.2%	Every day	3.5%
13 years old	34.6%	Once a week	1.8%
15 years old	33.2%	Less than once a week	2.7%
Gender		Don't	92.0%
Boy	49.2%	Alcopops	
Girl	50.8%	Every day	1.0%
Well off		Every week	1.4%
Very well off	19.5%	Every month	3.9%
Quite well off	34.4%	Rarely	12.6%
Average	39.0%	Never	81.0%
Not very well off	5.7%	TV (weekend)	
Not at all well off	1.4%	None at all	3.7%
TV (weekdays)		Half an hour a day	7.4%
None at all	5.5%	1 h a day	13.1%
Half an hour a day	14.5%	2 h a day	19.7%
1 h a day	21.6%	3 h a day	18.0%
2 h a day	23.5%	4 h a day	14.0%
3 h a day	15.6%	5 h a day	9.2%
4 h a day	8.6%	6 h a day	5.4%
5 h a day	4.6%	7 h or more a day	9.5%
6 h a day	1.9%	Play game (weekend)	
7 h or more a day	4.2%	None at all	13.7%
Play game (weekdays)		Half an hour a day	14.5%
None at all	19.0%	1 h a day	15.4%
Half an hour a day	20.2%	2 h a day	15.3%
1 h a day	19.2%	3 h a day	11.7%
2 h a day	15.6%	4 h a day	9.0%
3 h a day	9.7%	5 h a day	6.4%
4 h a day	5.9%	6 h a day	4.3%
5 h a day	3.7%	7 h or more a day	9.7%
6 h a day	1.9%	Computer (weekend)	
7 h or more a day	4.8%	None at all	9.6%
Computer (weekdays)		Half an hour a day	14.2%
None at all	10.1%	1 h a day	16.3%
Half an hour a day	19.2%	2 h a day	15.7%
1 h a day	20.4%	3 h a day	12.1%
2 h a day	16.8%	4 h a day	9.1%
3 h a day	11.1%	5 h a day	6.7%
4 h a day	7.1%	6 h a day	4.7%
5 h a day	4.9%	7 h or more a day	11.6%
6 h a day	2.8%	Fruits	
7 h or more a day	7.6%	Never	3.0%
Breakfast weekdays		Less than once a week	6.0%
Never	15.7%	Once a week	9.5%
One day	4.0%	2–4 days a week	28.2%
Two days	4.9%	5–6 days a week	15.4%
Three days	6.6%	Once daily	17.8%
Four days	5.5%	More than once daily	20.2%
Five days	63.3%	Sweets	
Breakfast weekend		Never	4.1%
Never	7.1%	Less than once a week	12.1%
One day	13.3%	Once a week	19.2%
Both days	79.5%	2–4 days a week	28.2%
Vegetables		5–6 days a week	12.8%
Never	4.5%	Once daily	12.0%
Less than once a week	5.8%	More than once daily	11.6%
Once a week	9.8%	Soft drinks	
2–4 days a week	25.3%	Never	11.1%
5–6 days a week	19.1%	Less than once a week	22.0%
Once daily	19.0%	Once a week	18.8%
More than once daily	16.6%	2–4 days a week	21.7%
Self-rated Health		5–6 days a week	8.7%
Excellent	36.4%	Once daily	7.4%
Good	50.2%	More than once daily	10.4%
Fair	11.8%	Frequency of vigorous physical activity	
Poor	1.6%	Every day	19.2%
Academic achievement		4–6 times a week	24.4%
Very good	24.0%	2–3 times a week	29.2%
Good	43.2%	Once a week	13.1%
Average	28.1%	Once a month	3.8%
Below average	4.7%	Less than once a month	4.7%

Table 2 presents the association between the frequency of vigorous physical activity and academic achievement. Results show that engaging in vigorous physical activity 4–6 times a week, 2–3 times a week, or once a week were associated with higher likelihood of lower academic achievement compared to the reference group, with OR of 1.03 (95% CI: 1.01–1.06), 1.03 (95% CI: 1.00–1.06), and 1.04 (95% CI: 1.01–1.08) respectively. Engaging in frequency of vigorous physical activity once a month showed OR of 0.96 (95% CI: 0.92–1.01), while engaging less than once a month or never showed OR of 1.09 (95% CI: 1.05–1.14) and 1.07 (95% CI: 1.03–1.12) respectively, indicating a slightly higher likelihood of lower academic achievement compared to the reference group.

**Table 2:** Association between the frequency of vigorous physical activity and academic achievement.

Frequency of vigorous physical activity	Academic achievement	
	OR	95% CI
Every day	Reference group	
4–6 times a week	**1** **.** **03**	**1.01–1.06**
2–3 times a week	**1**.**03**	**1.00–1.06**
Once a week	**1**.**04**	**1.01–1.08**
Once a month	0.96	0.92–1.01
Less than once a month	**1**.**09**	**1.05–1.14**
Never	**1**.**07**	**1.03–1.12**

OR, Odd Ratio; CI, Confidence Interval. Bold values: *p* < 0.05.

Model controlled for all covariates (age, gender, well off, self-rated health, screen time, eating habits, alcopops consumption, and smoking).

Table 3 illustrates the association between the frequency of vigorous physical activity and academic achievement in boys. Results indicate that there was no in general significant association between engaging in vigorous physical activity 4–6 times a week, 2–3 times a week, or once a week and academic achievement, as evidenced by OR of 1.02 (95% CI: 0.99–1.06), 1.00 (95% CI: 0.97–1.03), and 0.98 (95% CI: 0.93–1.02), respectively. Similarly, engaging in vigorous physical activity once a month, less than once a month, or never did not have significant associations with academic achievement, as indicated by OR of 0.93 (95% CI: 0.86–1.00), 1.02 (95% CI: 0.95–1.10), and 0.98 (95% CI: 0.92–1.05) respectively.

**Table 3:** Association between the frequency of vigorous physical activity and academic achievement in boys.

Frequency of vigorous physical activity	Academic achievement	
	OR	95% CI
Every day	Reference	
4–6 times a week	1.02	0.99–1.06
2–3 times a week	1.00	0.97–1.03
Once a week	0.98	0.93–1.02
Once a month	0.93	0.86–1.00
Less than once a month	1.02	0.95–1.10
Never	0.98	0.92–1.05

OR, Odd Ratio; CI, Confidence Interval.

Model controlled for all covariates (age, gender, well off, self-rated health, screen time, eating habits, alcopops consumption, and smoking).

Table 4 presents the relationship between the frequency of vigorous physical activity and academic achievement in girls. The results indicate a significant association between decreasing frequencies of vigorous physical activity and higher odds of lower academic achievement. Engaging in vigorous physical activity 4–6 times a week, 2–3 times a week, and once a week were associated with progressively higher odds of lower academic achievement, with OR of 1.06 (95% CI: 1.02–1.10), 1.08 (95% CI: 1.04–1.12), and 1.12 (95% CI: 1.08–1.17) respectively. Moreover, engaging in vigorous physical activity once a month, less than once a month, or never is also associated with higher probability of lower academic achievement, as indicated by OR of 1.02 (95% CI: 0.96–1.08), 1.18 (95% CI: 1.11–1.25), and 1.18 (95% CI: 1.11–1.24) respectively.

**Table 4:** Association between the frequency of vigorous physical activity and academic achievement in girls.

Frequency of vigorous physical activity	Academic achievement	
	OR	95% CI
Every day	Reference group	
4–6 times a week	**1** **.** **06**	**1.02–1.10**
2–3 times a week	**1**.**08**	**1.04–1.12**
Once a week	**1**.**12**	**1.08–1.17**
Once a month	1.02	0.96–1.08
Less than once a month	**1**.**18**	**1.11–1.25**
Never	**1**.**18**	**1.11–1.24**

OR, Odd Ratio; CI, Confidence Interval. Bold values: *p* < 0.05.

Model controlled for age, gender, well off, self-rated health, screen time, eating habits, alcopops consumption, and smoking.

## 4 Discussion

The current study presents the results on association between frequency of vigorous physical activity and academic achievement, aligning with previous findings while also highlighting some differences and consistencies. Consistent with prior research, the current study found a potential association between more vigorous physical activity and better academic achievement. This aligns with a growing body of literature that has demonstrated the positive effects of regular physical activity on cognitive function, attention, and academic performance. The current study's results indicate a potential association between vigorous physical activity and academic achievement (41, 42), especially in girls but not in boys. This finding presents a notable difference from the general trend observed in previous research, which has often shown positive associations between physical activity and academic achievement across genders (43, 44).

### 4.1 Association between vigorous physical activity and academic achievement

The significant associations observed in the current study are consistent with a substantial body of evidence indicating that physical activity is beneficial for cognitive function and academic performance (17, 45). Research has suggested that regular physical activity can lead to improvements in attention, memory, and information processing, all of which are fundamental to academic success (46, 47). These findings underscore the potential role of physical activity in enhancing overall academic achievement. The results also underscore the importance of considering potential confounding variables, which is consistent with previous findings (20, 48). As noted in the prior studies, it is essential to recognize that adolescents who engage in vigorous physical activity more frequently may have less time available for academic studying, potentially impacting their academic performance. This highlights the need to account for factors such as time allocation for studying, socioeconomic status, access to resources, nutrition and overall lifestyle habits, which have been consistently identified as potential moderators in the association between physical activity and academic achievement. The significant impact of breakfast consumption on nutritional benefits has been thoroughly demonstrated across various age groups, particularly among individuals engaged in mental and physical activities (49). Moreover, this study's findings prompt the need for further research to better understand the mechanisms underlying the association between vigorous physical activity and academic achievement (48, 50). This aligns with previous calls for more in-depth exploration into how different types of physical activity might impact cognitive function, attention, and overall academic performance. Understanding the specific mechanisms through which physical activity influences cognitive function and academic achievement is crucial for developing targeted interventions and strategies to support students’ overall well-being and academic success.

Engaging in vigorous physical activity has been demonstrated to exert profound effects on various aspects of cognitive functioning, particularly executive functions that can benefit academic success. The enhancement of working memory and cognitive flexibility, key components in problem-solving and comprehension tasks, underscores the significance of vigorous physical activity in fostering cognitive development during adolescence (51). The puberty period, characterized by dynamic neural changes, is particularly sensitive to the positive impact of vigorous physical activity. Research indicates that vigorous physical activity promotes neurogenesis, the generation of new neurons, and angiogenesis, the formation of new blood vessels. These neurobiological processes contribute to cognitive and mental health improvements, providing a solid foundation for effective learning. Moreover, vigorous physical activity plays a pivotal role in improving cardiorespiratory fitness (CRF), a factor intricately linked to cognitive performance. The positive association between CRF and cognitive abilities highlights the holistic benefits of vigorous physical activity on both physical and mental well-being. Adolescents engaging in regular vigorous physical activity not only experience improvements in their cardiovascular health but also enjoy enhanced cognitive functioning, ultimately supporting their academic endeavors. Additionally, vigorous physical activity exerts a positive impact on mental health, contributing to improved concentration and heightened engagement in academic activities. The psychological benefits of vigorous physical activity extend beyond the physiological realm, fostering a conducive environment for effective learning. This dual influence on cognitive and mental health aspects reinforces the notion that vigorous physical activity serves as a comprehensive strategy for promoting overall well-being in the academic context. Furthermore, the immediate cognitive benefits observed after vigorous physical activity, such as enhanced math test performance shortly following activity, underscore the direct and acute positive effects of vigorous physical activity on learning. The rapid improvements in cognitive functions provide valuable insights into the potential for incorporating vigorous physical activity as a proactive approach to enhance academic achievement.

### 4.2 Association between vigorous physical activity and academic achievement by gender

The current study finding suggests that a potential association between frequent vigorous physical activity and academic achievement particularly in girls. Previous research has consistently demonstrated the positive impact of physical activity on cognitive function, attention, and academic achievement across genders. However, the gender-specific nature of the association observed in the current study highlights the need to delve deeper into potential gender-related differences in how physical activity influences academic outcomes (43, 44). The positive association observed in girls aligns with a growing body of evidence that links physical activity to improved cognitive function and academic performance. Regular physical activity has been associated with enhanced attention, memory, and information processing, all of which are critical for academic success. The current study's findings add to the body of evidence, suggesting that vigorous physical activity may have a particularly beneficial impact on academic achievement in girls. This discrepancy underscores the need to further investigate potential gender-specific factors that may underlie the differing effects of physical activity on academic performance in boys and girls. Understanding why the association between vigorous physical activity and academic achievement appears to be specific to girls is crucial. Further research is needed to explore potential underlying mechanisms that may contribute to this gender-specific difference (52, 53). Factors such as hormonal influences, social dynamics, and differences in the ways boys and girls engage in physical activity could all play a role in shaping this relationship (54). The current study's findings emphasize the importance of considering potential confounding variables, such as time allocation for studying, socioeconomic status, access to resources, and overall lifestyle habits (20, 48). These factors may interact with physical activity in complex ways, influencing academic achievement differently in boys and girls.

The observed gender-specific effects in the context of physical activity and academic achievement may be attributed to the distinct patterns of vigorous physical activity across girls and boys, potentially exerting a more pronounced impact on cognitive function and academic success compared to the patterns observed in boys (52, 54). This distinction arises from variations in activity types, intensity, and duration, which could lead to differing cognitive and academic outcomes (1, 55). The study's findings in turn shed light on the potential influence of gender-specific physical activity patterns on cognitive and academic development. Moreover, the results of the study may be influenced by how boys and girls allocate their time between physical activity and academic pursuits (48). It is plausible that the time devoted to vigorous physical activity impacts academic engagement and study time differently for boys and girls, ultimately shaping their academic achievement in different ways. This suggests that the relationship between physical activity and academic success is mediated by gender-specific time management and engagement in academic activities. Understanding these differences in time allocation and activity engagement is crucial in comprehending the nuanced relationship between physical activity and academic performance across genders. Furthermore, the unique developmental changes associated with puberty in boys and girls may interact with physical activity in distinct ways, potentially influencing cognitive function and academic performance differently across genders (51). The onset of puberty brings about hormonal fluctuations and neurological developments that differ between boys and girls. These biological and developmental factors could contribute to the observed gender-specific effects, emphasizing the need to consider the impact of puberty-related changes on the relationship between physical activity and academic achievement (56, 57). In essence, the varying impact of physical activity on cognitive function and academic achievement between boys and girls underscores the importance of considering gender-specific activity patterns, time allocation, and developmental changes. These factors play a pivotal role in shaping the relationship between physical activity and academic success.

### 4.3 Strength and limitations

This study offers valuable insights into the association between vigorous physical activity and academic achievement in a substantial sample of adolescents. This study used data from 214,808 adolescents, offering a robust and representative sample for analysis. Also, this study incorporates various confounding variables, such as socioeconomic status, parental education, and school environment, into the statistical analyses, enhancing the internal validity of the association between vigorous physical activity and academic achievement. However, it is important to acknowledge the study limitations. The study findings primarily derive from data collected from adolescents in Europe and North America. There might be biases in the research findings due to regional and cultural differences. Cross-sectional design, this study used, limits the establishment of causal relationships between vigorous physical activity and academic achievement, as it only captures associations at a single point in time. Longitudinal studies are essential to explore the enduring effects of vigorous physical activity on academic performance. Self-reported variables like academic achievement and vigorous physical activity may introduce recall or reporting biases, potentially leading to overestimation or underestimation. Moreover, Biddle et al. (58) indicated that the vigorous physical activity item from the HBSC study demonstrates strong reliability and validity. However, other studies have noted that its validity compared to objective monitoring of physical activity is lacking, suggesting the possibility of errors stemming from subjective surveys. Although the study accounts for various covariates, there may still be unmeasured confounding factors that could impact the observed relationship between physical activity and academic performance.

### 4.4 Implications for research and practice

Future research should adopt longitudinal approaches to track changes over time and gain deeper insights into the association between vigorous physical activity and academic performance. Objective measures such as wearable devices and standardized tests could mitigate biases associated with self-reported measurement. Moreover, Future research could investigate this relationship in various geographical and cultural settings to improve the external validity of the findings. Furthermore, thorough exploration of gender differences in the impact of vigorous physical activity on academic achievement is warranted. Understanding the underlying mechanisms driving these differences is crucial for providing valuable insights into gender-specific effects. Therefore, future studies should delve deeper into these mechanisms to contribute to a more comprehensive understanding of the interplay between vigorous physical activity and academic performance.

## 5 Conclusions

The findings highlight the potential benefits of vigorous physical activity on academic achievement among adolescents in adolescents. Future research should be suggested to employ longitudinal designs, utilize objective measures of vigorous physical activity and academic achievement, conduct intervention studies, and explore mediating factors.

## Data Availability

Publicly available datasets were analyzed in this study. This data can be found here: Data from the Health Behaviour in School-aged Children (HBSC) survey.

## References

[1] Janssen I Leblanc AG. Systematic review of the health benefits of physical activity and fitness in school-aged children and you. Int J Behav Nutrit Phys Act. (2010) 7:40. 10.1186/1479-5868-7-40 20459784 PMC2885312

[2] Álvarez-Bueno C Pesce C Cavero-Redondo I Sánchez-López M Martínez-Hortelano JA Martínez-Vizcaíno V. The effect of physical activity interventions on children’s cognition and metacognition: a systematic review and meta-analysis. J Am Acad Child Adolesc Psychiatry. (2017) 56(9):729–38. 10.1016/j.jaac.2017.06.012 28838577

[3] Cöster M. The Effects of Increased Physical Activity in Childhood on Fracture Risk, Musculoskeletal Development, and Academic Achievement. Lund: Lund University (2017).

[4] Fritz J. Physical Activity During Growth. Effects on Bone, Muscle, Fracture Risk and Academic Performance. Lund: Lund University: Faculty of Medicine (2017).

[5] Mavilidi MF Drew R Morgan PJ Lubans DR Schmidt M Riley N. Effects of different types of classroom physical activity breaks on children’s on-task behaviour, academic achievement and cognition. Acta Paediatr. (2020) 109(1):158–65. 10.1111/apa.14892 31168863

[6] Mavilidi MF Vazou S. Classroom-based physical activity and math performance: integrated physical activity or not? Acta Paediatr. (2021) 110(7):2149–56. 10.1111/apa.15860 33780563

[7] Chaddock L Erickson KI Prakash RS Vanpatter M Kramer AF. Basal ganglia volume is associated with aerobic fitness in preadolescent children. Dev Neurosci. (2010) 32(3):249–56. 10.1159/000316648 20693803 PMC3696376

[8] Colcombe SJ Erickson KI Scalf PE Kim JS Prakash R Mcauley E Aerobic exercise training increases brain volume in aging humans. J Gerontol A Biol Sci Med Sci. (2006) 61(11):1166–70. 10.1093/gerona/61.11.1166 17167157

[9] Lardon MT Polich J. EEG changes from long-term physical exercise. Biol Psychol. (1996) 44(1):19–30. 10.1016/S0301-0511(96)05198-8 8906355

[10] Cotman CW Berchtold NC. Exercise: a behavioral intervention to enhance brain health and plasticity. Trends Neurosci. (2002) 25(6):292–301. 10.1016/S0166-2236(02)02143-4 12086747

[11] Chaddock-Heyman L Erickson KI Kienzler C King M Pontifex MB Raine LB The role of aerobic fitness in cortical thickness and mathematics achievement in preadolescent children. PloS One. (2015) 10(8):e0134115. 10.1371/journal.pone.0134115 26267897 PMC4534422

[12] Schaeffer DJ Krafft CE Schwarz NF Chi L Rodrigue AL Pierce JE An 8-month exercise intervention alters frontotemporal white matter integrity in overweight children. Psychophysiology. (2014) 51(8):728–33. 10.1111/psyp.12227 24797659 PMC4107135

[13] Chaddock L Hillman CH Pontifex MB Johnson CR Raine LB Kramer AF. Childhood aerobic fitness predicts cognitive performance one year later. J Sports Sci. (2012) 30(5):421–30. 10.1080/02640414.2011.647706 22260155

[14] Hillman CH Castelli DM Buck SM. Aerobic fitness and neurocognitive function in healthy preadolescent children. Med Sci Sports Exerc. (2005) 37(11):1967–74. 10.1249/01.mss.0000176680.79702.ce 16286868

[15] Monti JM Hillman CH Cohen NJ. Aerobic fitness enhances relational memory in preadolescent children: the FITKids randomized control trial. Hippocampus. (2012) 22(9):1876–82. 10.1002/hipo.22023 22522428 PMC3404196

[16] Moore RD Wu CT Pontifex MB O'Leary KC Scudder MR Raine LB Aerobic fitness and intra-individual variability of neurocognition in preadolescent children. Brain Cogn. (2013) 82(1):43–57. 10.1016/j.bandc.2013.02.006 23511845 PMC3632076

[17] Sibley B Etnier J. The relationship between physical activity and cognition in children: a meta-analysis. Pediatr Exerc Sci. (2003) 15(3):243–56. 10.1123/pes.15.3.243

[18] Donnelly JE Hillman C Castelli D Etnier JL Szabo-Reed A. Physical activity, fitness, cognitive function, and academic achievement in children: a systematic review. Med Sci Sports Exerc. (2016) 48(6):1197–222. 10.1249/MSS.0000000000000901 27182986 PMC4874515

[19] Esteban-Cornejo I Tejero-Gonzalez CM Sallis JF Veiga OL. Physical activity and cognition in adolescents: a systematic review. J Sci Med Sport. (2014) 18(5):534–9. 10.1016/j.jsams.2014.07.007 25108657

[20] Rasberry CN Lee SM Robin L Laris BA Russell LA Coyle KK The association between school-based physical activity, including physical education, and academic performance: a systematic review of the literature. Prev Med. (2011) 52(supp-S):S10–20. 10.1016/j.ypmed.2011.01.027 21291905

[21] Fedewa AL Ahn S. The effects of physical activity and physical fitness on children’s achievement and cognitive outcomes: a meta-analysis. Res Q Exerc Sport. (2011) 82(3):521–35. 10.1080/02701367.2011.10599785 21957711

[22] Verburgh L Konigs M Scherder EJA Oosterlaan J. Physical exercise and executive functions in preadolescent children, adolescents and young adults: a meta-analysis. Br J Sports Med. (2014) 48(12):973. 10.1136/bjsports-2012-091441 23467962

[23] Schmidt M Jaeger K Egger F Roebers CM Conzelmann A. Cognitively engaging chronic physical activity, but not aerobic exercise, affects executive functions in primary school children: a group-randomized controlled trial. J Sport Exerc Psychol. (2015) 37(6):575–91. 10.1123/jsep.2015-0069 26866766

[24] Altenburg TM Chinapaw MJ Singh AS. Effects of one versus two bouts of moderate intensity physical activity on selective attention during a school morning in Dutch primary schoolchildren: a randomized controlled trial. J Sci Med Sport. (2016) 19(10):820–4. 10.1016/j.jsams.2015.12.003 26724833

[25] Hillman CH Pontifex MB Castelli DM Khan NA Raine LB Scudder MR Effects of the FITKids randomized controlled trial on executive control and brain function. Pediatrics. (2014) 134(4):e1063–71. 10.1542/peds.2013-3219 25266425 PMC4179093

[26] Hansen DM Herrmann SD Lambourne K Lee J Donnelly JE. Linear/nonlinear relations of activity and fitness with children’s academic achievement. Med Sci Sports Exerc. (2014) 46(12):2279–85. 10.1249/MSS.0000000000000362 24781896 PMC4211996

[27] Jaakkola T Hillman C Kalaja S Liukkonen J. The associations among fundamental movement skills, self-reported physical activity and academic performance during junior high school in Finland. J Sports Sci. (2015) 33(16):1719–29. 10.1080/02640414.2015.1004640 25649279

[28] Tremblay MS Inman JW Willms JD. The relationship between physical activity, self-esteem, and academic achievement in 12-year-old children. Pediatr Exerc Sci. (2000) 12(3):312–23. 10.1123/pes.12.3.312

[29] Costigan SA Eather N Plotnikoff RC Hillman C H.Lubans DR. High-intensity interval training for cognitive and mental health in adolescents. Med Sci Sports Exercise. (2016) 48(10):1985–93. 10.1249/MSS.0000000000000993 27187097

[30] Jeon YK Ha CH. The effect of exercise intensity on brain derived neurotrophic factor and memory in adolescents. Environ Health Prev Med. (2017) 22(1):27. 10.1186/s12199-017-0643-6 29165142 PMC5664787

[31] Moreau D Kirk IJ Waldie KE. High-intensity training enhances executive function in children in a randomized, placebo-controlled trial. eLife Sci. (2017) 6:e25062. 10.7554/eLife.25062 28825973 PMC5566451

[32] Chang YK Labban JD Gapin JI Etnier JL. The effects of acute exercise on cognitive performance: a meta-analysis. Brain Res. (2012) 1453:87–101. 10.1016/j.brainres.2012.02.068 22480735

[33] Castelli DM Hillman CH Hirsch J Hirsch A Drollette E. FIT Kids: time in target heart zone and cognitive performance. Prev Med. (2011) 52(Suppl 1):S55–9. 10.1016/j.ypmed.2011.01.019 21281671

[34] Currie C Inchley J Molcho M Lenzi M Veselska Z Wild F. Health Behaviour in School-Aged Children (HBSC) Study Protocol: Background, Methodology and Mandatory Items for the 2013/14 Survey. St Andrews, UK: Child and Adolescent Health Research Unit (CAHRU) (2014).

[35] Roberts C Freeman J Samdal O Schnohr CW De Looze M Nic Gabhainn S The health behaviour in school-aged children (HBSC) study: methodological developments and current tensions. Int J Public Health. (2009) 54:140–50. 10.1007/s00038-009-5405-9 19639259 PMC2732766

[36] Su Y Zhang Y Chen S Hong J-T Wang H. Is the health behavior in school-aged survey questionnaire reliable and valid in assessing physical activity and sedentary behavior in young populations? a systematic review. Front Public Health. (2022) 10:729641. 10.3389/fpubh.2022.729641 35419332 PMC8995780

[37] Booth ML Okely AD Chey T Bauman A. The reliability and validity of the physical activity questions in the WHO health behaviour in schoolchildren (HBSC) survey: a population study. Br J Sports Med. (2001) 35(4):263–7. 10.1136/bjsm.35.4.263 11477024 PMC1724349

[38] Rangul V Holmen TL Kurtze N Cuypers K Midthjell K. Reliability and validity of two frequently used self-administered physical activity questionnaires in adolescents. BMC Med Res Methodol. (2008) 8:47. 10.1186/1471-2288-8-47 18627632 PMC2492874

[39] Bobakova D Hamrik Z Badura P Sigmundova D Nalecz H Kalman M. Test-retest reliability of selected physical activity and sedentary behaviour HBSC items in the Czech republic, Slovakia and Poland. Int J Public Health. (2015) 60(1):59–67. 10.1007/s00038-014-0628-9 25471078

[40] Zhang D Hong J Chen S Liu Y. Associations of physical activity with academic achievement and academic burden in Chinese children and adolescents: do gender and school grade matter? BMC Public Health. (2022) 22(1):1496. 10.1186/s12889-022-13886-3 35932047 PMC9356485

[41] Trudeau F Shephard RJ. Physical education, school physical activity, school sports and academic performance. Int J Behav Nutrit Phys Act. (2008) 5:10. 10.1186/1479-5868-5-10 18298849 PMC2329661

[42] Coe DP Peterson T Blair C Schutten MC Peddie H. Physical fitness, academic achievement, and socioeconomic status in school-aged youth. J School Health. (2013) 83(7):500–7. 10.1111/josh.12058 23782093

[43] Kristensen PL Korsholm L C.Møller N Wedderkopp N Andersen LB Froberg K. Sources of variation in habitual physical activity of children and adolescents: the European youth heart study. Scand J Med Sci Sports. (2010) 18(3):298–308. 10.1111/j.1600-0838.2007.00668.x 17555541

[44] Pate RR Trost SG Levin S Dowda M. Sports participation and health-related behaviors among US youth. Arch Pediatr Adolesc Med. (2000) 154(9):904–11. 10.1001/archpedi.154.9.904 10980794

[45] Hillman CH Erickson KI Kramer AF. Be smart, exercise your heart: exercise effects on brain and cognition. Nat Rev Neurosci. (2008) 9(1):58–65. 10.1038/nrn2298 18094706

[46] Tomporowski PD Davis CL Naglieri MJA. Exercise and children’s intelligence, cognition, and academic achievement. Educ Psychol Rev. (2008) 20(2):111–31. 10.1007/s10648-007-9057-0 19777141 PMC2748863

[47] Best JR. Effects of physical activity on children’s executive function: contributions of experimental research on aerobic exercise. Dev Rev. (2010) 30(4):331–551. 10.1016/j.dr.2010.08.001 21818169 PMC3147174

[48] Singh AS Saliasi E Vvd B Uijtdewilligen L Groot R Jolles J Effects of physical activity interventions on cognitive and academic performance in children and adolescents: a novel combination of a systematic review and recommendations from an expert panel. Br J Sports Med. (2019) 53(10):640–7. 10.1136/bjsports-2017-098136 30061304

[49] Taheri M Irandoust K Seghatoleslamy A Eshaghi S Valayi F. The acute effect of breakfast cereal consumption on inhibitory cognitive control in competitive male collegiate athlete’s with habitual breakfast skipping. Biol Rhythm Res. (2021) 52(5):774–80. 10.1080/09291016.2019.1603694

[50] Trudeau F Shephard RJ. Relationships of physical activity to brain health and the academic performance of schoolchildren. Am J Lifestyle Med. (2010) 4(2):138–50. 10.1177/1559827609351133

[51] Mabounda Kounga P Eddie Janvier B Nsompi F Kayilou JM Nguimbi E Zhang Y Influences of puberty development and sexuality on academic performance among secondary students of ngamaba tsalakoua of Brazzaville (Republic of Congo). Int J Sport Stud Health. (2023) 5(2):e133815. 10.5812/intjssh-133815

[52] Carlson SA Fulton JE Lee SM Maynard LM Brown DR Kohl HW Physical education and academic achievement in elementary school: data from the early childhood longitudinal study. Am J Public Health. (2008) 98(4):721–7. 10.2105/AJPH.2007.117176 18309127 PMC2377002

[53] Slater A Tiggemann M. Gender differences in adolescent sport participation, teasing, self-objectification and body image concerns. J Adolesc. (2011) 34(3):455–63. 10.1016/j.adolescence.2010.06.007 20643477

[54] Ekeland E Heian F Hagen RB Abbott J Nordheim L. Exercise to improve self-esteem in children and young people. Campbell Syst Rev. (2005) 1:CD003683. 10.4073/csr.2005.4 PMC12935395 14974029

[55] Strong WB Malina RM Blimkie CJ Daniels SR Dishman RK Gutin B Evidence based physical activity for school-age youth. J Pediatr. (2005) 146(6):732–7. 10.1016/j.jpeds.2005.01.055 15973308

[56] Berkey CS Rockett HRH Field AE Gillman MW Frazier AL Camargo CA Activity, dietary intake, and weight changes in a longitudinal study of preadolescent and adolescent boys and girls. Pediatrics. (2000) 105(4):E56. 10.1542/peds.105.4.e56 10742377

[57] Ge X Conger RD Elder GH. Pubertal transition, stressful life events, and the emergence of gender differences in adolescent depressive symptoms. Dev Psychol. (2001) 37(3):404–17. 10.1037/0012-1649.37.3.404 11370915

[58] Biddle SJ Gorely T Pearson N Bull FC. An assessment of self-reported physical activity instruments in young people for population surveillance: project ALPHA. Int J Behav Nutr Phys Act. (2011) 8:1. 10.1186/1479-5868-8-1 21194492 PMC3019119

